# Elevated risk of depression and anxiety disorder by „high strain“ occupations: a systematic review

**DOI:** 10.1093/eurpub/ckac130.091

**Published:** 2022-10-25

**Authors:** A Seidler, A Freiberg, S Drössler, FS Hussenoeder, I Conrad, S Riedel-Heller, K Romero Starke, M Schubert

**Affiliations:** 1 TU Dresden, Faculty of Medicine, Institute of Occupational and Social Medicine, Dresden, Germany; 2 University of Leipzig, Institute of Social Medicine, Occupational Occupational Medicine and Public Health, Leipzig, Germany

## Abstract

**Background:**

Poor working conditions might lead to mental illness.

**Methods:**

We performed a systematic review with meta-analyses as an update of a review published in 2013. We registered the study protocol with PROSPERO (registration number: CRD42020170032) and searched for epidemiological studies in MEDLINE, PsycINFO, and Embase. Two reviewers carried out independently all review steps including title-abstract screening, full-text screening, risk-of-bias assessment and data extraction. Discordances were solved by consensus. We determined the certainty of evidence using the GRADE-approach.

**Results:**

Ten cohort studies with acceptable study quality examined the relationship between high job strain and the incidence of depression. In the “classic” demand-control-model, ‘high strain’ (combination of high demands and low control) is compared with ‘low strain’ (combination of low demands and high job control). For high strain, the risk of depression was elevated by 73%, the pooled effect estimate for the risk of depression was 1.73 (95% CI 1.32-2.27. In a dichotomous analysis (without dividing job strain into the four dimensions mentioned above), there was a doubled risk of depression with high job strain (pooled effect estimate=1.99, 95% CI 1.68-2.35). We found comparable risk estimates for men and women. The GRADE assessment revealed a high certainty of evidence of the association between job strain and depression. We also found a considerably increased risk of anxiety disorder among individuals prone to high job strain.

**Conclusions:**

This systematic review finds a clear association between high job strain (high demands in combination with low control) and depression as well as anxiety disorders.

Acknowledgment: This study was financially supported by SUVA (Schweizerische Unfallversicherungsanstalt).

**Key messages:**

- High job strain (high demands, low control) is clearly associated with depression and anxiety disorders.
- The GRADE assessment revealed a high certainty of evidence of the association between job strain and depression.

